# A Dreaded Complication of Corrosive Poisoning Leading to Gangrene of the Stomach and Duodenum: A Rare Case

**DOI:** 10.7759/cureus.60313

**Published:** 2024-05-14

**Authors:** Chandrashekar Patil, V. M. Shankar Reddy, Raavi Ravindranadh, Deepa Bommanagari, Dhruv R. P.

**Affiliations:** 1 Radiology, Mallareddy Medical College for Women, Hyderabad, IND; 2 Surgery, Mallareddy Medical College for Women, Hyderabad, IND; 3 General Surgery, Mallareddy Medical College for Women, Hyderabad, IND; 4 Radiodiagnosis, Mallareddy Medical College for Women, Hyderabad, IND

**Keywords:** gastric outlet obstruction, edematous pylorus, coagulative necrosis, impending perforation, corrosive poisoning

## Abstract

Corrosive poisoning is common in developing countries like India. It is mainly due to accidental consumption in children, whereas suicide is the usual intent in adults. It leads to devastating injuries, to the upper gastrointestinal tract such as necrosis and perforation. The long-term complications include stricture formation and gastric outlet obstruction. Here, we present the case of a 50-year-old male with an alleged history of corrosive acid ingestion. On contrast-enhanced computed tomography (CECT) of the abdomen, there was an absence of wall enhancement of the stomach and the first part of the duodenum, which was suggestive of necrosis or gangrenous changes with signs of impending perforation of the stomach and the first part of the duodenum. The patient was immediately taken up for surgery, and the intraoperative findings were consistent with the imaging findings.

## Introduction

In developing countries, corrosive injuries to the upper gastrointestinal tract are not uncommon [1]. Accidental consumption is more common in children, whereas suicide is the usual cause in adults [2]. Corrosives usually affect the upper gastrointestinal tract [3]. The degree, site, and extent of injury depend on the corrosive agent and duration of contact. Acids are known to induce greater damage to gastric mucosa, whereas alkalis are more likely to cause damage to esophageal mucosa [4]. Acidic agents commonly available are aqua regia and cleaning agents (hydrochloric acid) [5]. Commonly available alkalis are sodium and potassium hydroxides. Acidic and alkaline injuries can cause coagulation necrosis and liquefaction necrosis, respectively. Corrosive gastric injuries include a wide spectrum of acute mucosal (partial or total) or transmural involvement and different types of chronic injuries [1]. Radiological imaging helps evaluate the severity of injury.

## Case presentation

**Figure 1:**
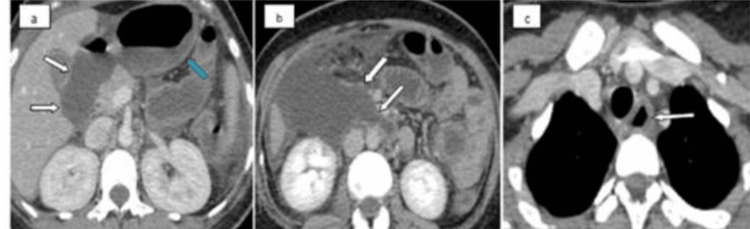
(a) CECT axial images showing severely edematous pylorus and the duodenum with absent wall enhancement suggestive of necrosis/ischemia (arrows). Note normal enhancement of the body of the stomach (blue arrow). (b) Absent enhancement of the duodenal wall. (c) Edematous cervical esophagus (arrow).

**Figure 2:**
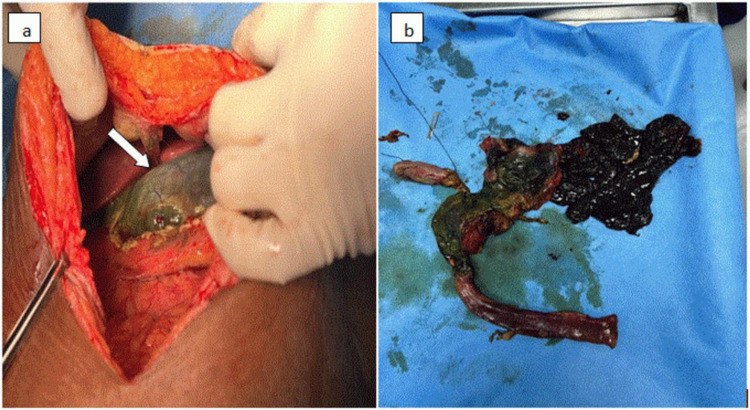
(a) Intraoperative images showing a gangrenous stomach. (b) Resected specimen of the gangrenous stomach, duodenum, and part of the jejunum.

A 26-year-old female presented to the emergency department with a history of ingestion of around 30 mL of toilet cleanser (20% hydrochloric acid). She gave a history of three episodes of blood vomiting with difficulty swallowing. A history of chest pain and abdominal pain was present. The patient was a known case of seizure disorder and was on regular medications. On examination, she was afebrile and conscious. Her vital signs were normal: blood pressure of 110/70 mmHg, pulse rate of 110 beats/min, respiratory rate of 20 breaths/min, saturation of peripheral oxygen of 98% on room air. On clinical examination, there was epigastric tenderness. Routine blood investigations revealed decreased hemoglobin level (5.9 g/dL), red blood cells (2.69 million/cu.mm), packed cell volume (20%), mean corpuscular volume (74.3 fL), mean corpuscular hemoglobin (21.9 pg), and mean corpuscular hemoglobin concentration (29.5 gm/dL), and increased white blood cells and red cell distribution width (18.5 fL). Chest X-ray revealed no evidence of any free air under the diaphragm and showed consolidation in the right lower lobe. An upper gastrointestinal (GI) endoscopy was performed on the day of ingestion, which revealed a Zarger grade IIIb esophageal injury and grade IIIb gastric injury. Abdominal contrast-enhanced computed tomography (CECT) revealed fluid-filled severely edematous pylorus and the first part of the duodenum with no delineation of the stomach wall and the first part of the duodenal wall (represents absent wall enhancement and thinning) with surrounding mild mesenteric fat stranding (Figures 1a, 1b); features were suggestive of corrosive ischemic injury to the stomach and the first part of the duodenal wall with possible potential impending perforation of the stomach and the first part of the duodenal wall. There was mild focal stricturous luminal narrowing seen at the distal second part of the duodenum with proximal upstream dilatation. Moderate ascites was seen in the bilateral paracolic gutters, right anterior pararenal space, and the pelvis with thin peritoneal enhancement. CECT of the chest revealed mild wall edema in the cervical esophagus (Figure 1c). A patch of consolidation was seen in the dependent segment of the right lower lobe, which was suggestive of severe aspiration pneumonitis. Based on the above imaging findings, the patient was taken for emergency surgery due to the high risk of upper gastrointestinal tract perforation. Intraoperatively, the stomach, duodenum, and a part of the jejunum showed complete gangrenous changes (Figures 2a, 2b), and hence surgical resection of the gangrenous viscera was performed with Whipple’s procedure, and a feeding jejunostomy was placed under general anesthesia. The post-operative period was uneventful, and the patient was treated with supportive medications. The patient was discharged in a hemodynamically stable condition and was allowed oral feeds at the time of discharge. She was doing well on regular follow-ups with no complications.

## Discussion

The extent of gastrointestinal injury depends on mucosal defenses, corrosive ingestion, and mode of consumption. In the acute phase, the severity ranges from mild injury to life-threatening perforation. In the chronic phase, stricture is frequent, resulting in difficulty in swallowing and malnutrition [6].

In the acute phase, arteriolar and venous thrombosis with consequent ischemia and mucosal sloughing is followed by bacterial invasion. Perforation occurs if an ulceration extends beyond the muscular plane [7]. Acids cause coagulative necrosis in tissues. The formation of the coagulum serves as a protective barrier, preventing the acid from penetrating further, thereby restricting the extent of injury [8]. The potential of acids to induce pylorospasm leads to the pooling of corrosive agents ingested in the prepyloric region, with a resultant rise in the time of contact of the corrosive agent with the pyloric mucosa and, therefore, the formation of strictures. In cases of high acid consumption, the stomach becomes necrotic, and gastric perforation occurs, which is a rare but potentially fatal complication. Alkalis are known to cause liquefactive necrosis, which leads to greater penetration and poses a high risk of injury to adjacent organs.

The initial imaging modalities used are chest and abdominal radiographs. The findings can include changes in aspiration pneumonitis, pneumothorax, pleural effusion, pneumomediastinum, and pneumoperitoneum.

Upper gastrointestinal endoscopy is usually performed to assess the grade of esophageal and gastric injuries. It can be safely performed up to four days after corrosive injury. The Zarger classification grades the severity of mucosal injury and helps guide management and gives prognostic information. The differentiation between superficial and transmural necrosis cannot be determined using endoscopy.

Computed tomography (CT) is the preferred diagnostic modality when there is a risk of or a known perforation. Using endoscopic correlation, Ryu et al. provided four grades of injuries: grade I, normal wall (thickness <3 mm); grade II, wall edema only (thickness >3 mm); grade III, wall edema with surrounding soft tissue stranding, with sharp interface; and grade IV, wall edema with surrounding soft tissue stranding and ill-defined interface with or without collection [9].

Chirica et al. proposed another classification, which determines viability of the gastric wall based on the presence and extent of wall enhancement on CT scan: grade I, normal-appearing organs; grade II, wall edema with surrounding soft tissue inflammation and post-contrast wall enhancement; and grade III, absence of post-contrast wall enhancement, suggesting transmural involvement [10].

CT scan also helps identify any associated findings in the chest and abdomen, such as aspiration pneumonitis, pneumoperitoneum, pleural effusion, and abdominal and mediastinal collections.

Management options include initial airway resuscitation and hemodynamic stabilization. During the acute phase, low-grade injuries are managed conservatively. Emergency surgery, including removal of gangrenous viscera, is indicated in cases of perforation and transmural involvement. In the chronic phase, strictures are managed by endoscopic stricture dilatation and stent placement. Surgical management is preferred in cases of multiple strictures, long segment strictures, and failure of endoscopic treatment [11].

Ushio reported a case of acute gastric necrosis in a patient who ingested a corrosive acid. The patient experienced hemodynamic shock on admission. Abdominal CECT revealed pneumoperitoneum, absent gastric wall enhancement, and free fluid in the abdomen. The patient underwent a laparotomy, which revealed transmural gastric necrosis and perforation. The patient underwent subtotal gastrectomy with Bill Roth I anastomosis. Post-operatively, the patient died due to septic shock [12].

## Conclusions

Acute corrosive poisoning injury to the upper gastrointestinal tract is a common condition in developing countries. Ingestion of corrosive chemical substances can result in devastating gastrointestinal tract injuries with the risk of perforation and/or hemorrhage. Gangrenous changes in the stomach and proximal small bowel are a rare and potentially fatal complication of acid ingestion. Familiarity with these imaging features of gangrenous changes in the bowel is very important in the diagnosis and hence helps the surgeon guide and timely intervene with the suitable management.
